# Anesthesia management in 14 cases of cesarean delivery in renal transplant patients—a single-center retrospective observational study

**DOI:** 10.1186/s40981-020-0317-z

**Published:** 2020-02-07

**Authors:** Shunsaku Goto, Risa Fukushima, Makoto Ozaki

**Affiliations:** 1 Department of Anesthesiology, Tokyo Women’s Medical University, Tokyo, Japan; 2 Department of Anesthesiology, Moriya Daiichi General Hospital, 1-17 Matsumaedai, Moriya-shi, Ibaraki, 302-0102 Japan

**Keywords:** Cesarean delivery, Renal transplant, Kidney, Anesthesia

## Abstract

**Background:**

The aim of this study was to investigate anesthesia management for cesarean delivery in renal transplant patients.

**Methods:**

The details of anesthesia management, patient characteristics, surgical information, and renal and maternal outcomes were retrospectively investigated in 14 post-renal transplant patients who underwent cesarean delivery at a single university hospital between January 1, 2014, and August 31, 2018.

**Results:**

Five patients were managed under general anesthesia, and nine cases were under regional anesthesia. Nine cases were emergency surgeries. The mean (SD) age was 35.5 (4.4) years, pregnancy body weight was 56.8 (10.0) kg, and gestational age was 33.3 (4.1) weeks. Nine cases were preterm deliveries. Five cases showed hypertension prior to pregnancy, and 13 patients showed hypertension before cesarean delivery. The preoperative creatinine level was 1.49 (0.53) mg/dL. The intraoperative maximum systolic/diastolic blood pressure was 170 (20)/102 (15) mmHg, and the intraoperative minimum systolic/diastolic blood pressure was 97 (13)/49 (12) mmHg. A total of six patients had either mean arterial pressure < 65 mmHg or required vasopressors. Serum creatinine remained unchanged after surgery compared with the preoperative level.

**Conclusion:**

Cesarean delivery was often performed in post-renal transplant patients for preterm delivery or as emergency surgery, with a higher ratio of regional anesthesia to general anesthesia. Since both hypertension and hypotension are most likely to occur during cesarean delivery, circulation management can be difficult, and anesthesia should be managed so as to maintain sufficient renal perfusion and ensure postoperative renal function.

## Background

Recently, the number of renal transplant patients who later give birth has increased significantly [1]. There have also been numerous reports on the potential perinatal complications of post-renal transplant pregnancy, the most common of which are hypertension, diabetes, preterm delivery, cesarean delivery, stillbirth, and miscarriage [2–4]. The higher rate of complications in such pregnancies is thought to be due to several factors, including impaired renal function prior to pregnancy, increased kidney stress due to a higher volume of blood plasma, compression of the transplanted kidney and ureter from the gravid uterus, and the negative effects of medications such as immunosuppressants on renal function.

In the long term, renal function in transplanted kidneys before and after pregnancy is similar to that in nonpregnant transplant recipients [5]; however, there is a short-term deterioration in renal function immediately before and after pregnancy [2]. In addition to the recognized increase in the number of renal transplant patients giving birth, cesarean delivery rates for this group are also particularly high, 56.9 to 62.6% [2, 3], so that anesthesiologists will be more likely to encounter such cases. Furthermore, although there are many reports on perinatal management and complications of cesarean delivery in renal transplant patients, there are relatively few reports on anesthesia [6, 7]. In a study on 64 cases of cesarean delivery for post-renal transplant patients, while the details of the anesthesia method and blood transfusion volumes were presented for 44 of the cases [6], there was no mention of the intraoperative hemodynamics. Examination of these hemodynamic characteristics is important for improving long-term surgical outcomes and maintaining postoperative renal function.

Therefore, we performed a retrospective study of anesthesia management for cesarean delivery in post-renal transplant patients with particular focus on the intraoperative hemodynamics depending on each anesthesia method.

## Methods

This retrospective observational study was carried out at a single teaching hospital and was reviewed and approved by the ethics committee of Tokyo Women’s Medical University Hospital (approval number 4328-R). As a retrospective observational study, informed consent was omitted and information about the study was published on the hospital website. The protocol was registered at the UMIN Clinical Trials Registry (No. UMIN000034406).

Cesarean deliveries from post-renal transplant patients at our institution between January 1, 2014, and August 31, 2018, were collected. Patients in whom dialysis was reintroduced prior to cesarean delivery were excluded. Cases that met the study criteria were examined regarding the following factors: (1) anesthesia information, including the anesthesia method and reasons for selection of the anesthesia method; (2) patient characteristics, including maternal age, height, pregnancy body weight, gestational age at time of surgery, emergency surgery, indications for cesarean delivery, hypertension (systolic pressure > 140 mmHg or diastolic pressure > 90 mmHg) before pregnancy or delivery, serum creatinine or complete blood count before pregnancy and cesarean delivery, time between renal transplant and delivery, original diseases that led to renal transplantation, and immunosuppressant usage and oral steroid usage; (3) surgical information, including operation time, operative fluid balance, perioperative hemodynamics, timing of hypotension, and use of vasopressors; and (4) renal and maternal outcomes, including kidney rejection or kidney injury after cesarean delivery and complications related to anesthesia. These items were collected from electronic medical records and the anesthesia management system Mirrel (Fukuda Denshi, Tokyo, Japan).

## Results

**Fig. 1:**
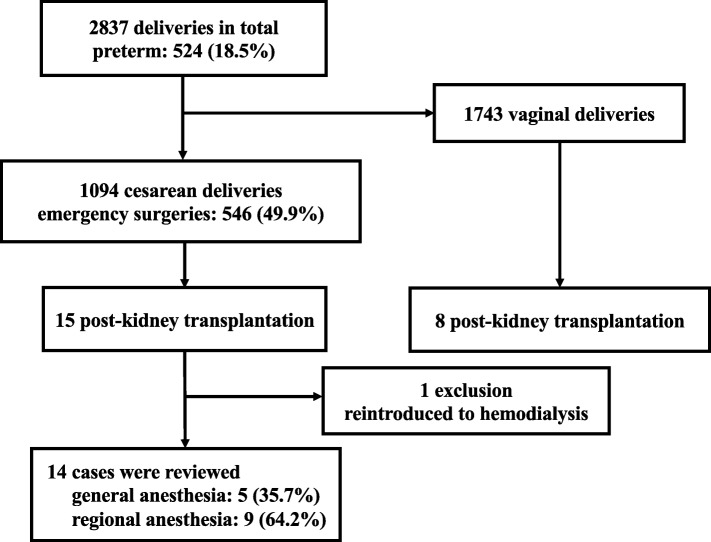
Patient selection flow chart in this study

The total number of deliveries was 2837, including 1094 cesarean deliveries (546 cases of emergency cesarean delivery) and 524 cases of preterm delivery (< 37 weeks). Among the 23 post-renal transplant cases, 15 parturients underwent cesarean deliveries. After excluding one case required reintroduction of dialysis before surgery, 14 cases were analyzed (Fig. 1).

Five cases received general anesthesia, and the remaining nine cases received regional anesthesia (four combined spinal-epidural and five spinal anesthesia). None of the cases under regional anesthesia was converted to general anesthesia. Among the 15 cases, nine cases were emergency cesarean deliveries (three general anesthesia, one combined spinal-epidural, and five spinal anesthesia). The reasons for selecting general anesthesia were as follows: urgent delivery due to prolonged deceleration (*n* = 1), spinal anesthesia not recommended due to low cerebrospinal fluid pressure syndrome (*n* = 1), and thrombocytopenia (100,000/mm3 or less) (*n* = 1), and to avoid spinal anesthesia due to cardiac dysfunction (*n* = 1) and ongoing immunosuppressants and type I diabetes (*n* = 1).

Patient characteristics are shown in Table 1. The most common reason for renal transplant was IgA nephropathy, and most of the patients received two immunosuppressants, tacrolimus, and azathioprine as well as corticosteroid, prednisolone, or methylprednisolone during pregnancy. No patients were accompanied with granulocytopenia or leucopenia by immunosuppressant therapy. Mean time to cesarean delivery after transplant was 5.2 (SD 3.7) years. Mean gestational age was older in patients receiving regional anesthesia than those receiving general anesthesia (34.7 vs 30.7 weeks), respectively, suggesting that general anesthesia was more likely chosen in preterm patients. The most common indication of cesarean delivery was hypertensive disorders of pregnancy (HDP) (*n* = 8).

**Table 1:** Patient characteristics

	Total	GA	RA
*N*	14	5	9
Age (years)	35.5 (4.4)	37.2 (5.2)	34.6 (3.6)
Height (cm)	156.6 (6.2)	157.8 (2.3)	156.0 (7.5)
Weight (kg)	56.8 (10.0)	58.0 (5.9)	46.1 (11.7)
Gestational weeks	33.3 (4.1)	30.7 (3.6)	34.7 (3.6)
Preterm (< 37 weeks)	9 (64.2%)	4 (80%)	5 (55.5%)
Emergency surgery	9 (64.2%)	3 (60%)	6 (66.7%)
Reason for CD (overlapping)			
Hypertensive disorders of pregnancy	8	4	4
Previous CD	3	0	3
NRFS	2	1	1
Dystocia	2	0	2
Placenta previa	1	0	1
Fetal growth restriction	1	0	1
Multiple pregnancy (twins)	1	0	1
Hypertension before pregnancy	5 (35.8%)	2 (40%)	3 (33.3%)
Hypertension before CD	13 (92.9%)	5 (100%)	8 (88.9%)
Preoperative creatinine (mg/dL)	1.49 (0.53)	1.88 (0.63)	1.27 (0.30)
Preoperative hemoglobin (g/dL)	9.6 (1.4)	9.5 (1.2)	9.6 (1.5)
Preoperative platelets (× 10^9^/L)	182 (61)	180 (61)	183 (62)
Preoperative white blood cells (× 10^9^/L)	9.3 (0.3)	10.6 (0.3)	8.6 (0.3)
Time between transplant and delivery	5.2 (3.7)	3.2 (2.3)	6.3 (3.8)
Reason for renal transplant			
IgA nephropathy	5	2	3
Type 1 diabetes	1	1	
Type 2 diabetes	1	1	
Lupus nephritis	1	1	
Focal glomerular nephritis	1		1
Interstitial nephritis	1		1
Hemolytic uremic syndrome	1		1
Hypertensive disorders of pregnancy	1		1
Hypovolemic shock	1		1
Unknown	1		1
Antirejection medication			
Tacrolimus	13	4	9
Azathioprine	13	5	8
Cyclosporine	1	1	0
Methylprednisolone or prednisolone	12	3	9

Values are mean (SD) or number (%)

*GA* general anesthesia, *RA* regional anesthesia, *CD* cesarean delivery, *NRFS* non-reassuring fetal status

Five cases showed hypertension (systolic blood pressure > 140 mmHg or diastolic blood pressure > 90 mmHg) prior to pregnancy. All patients had hypertension except one receiving regional anesthesia before cesarean delivery. Preoperative creatinine level was 1.49 (0.53) (SD) mg/dL.

The details of surgical information are shown in Table 2. A total of six patients (43%) had either mean blood pressure < 65 mmHg with the mean duration of 4 min or required vasopressors. The details of six patients with intraoperative hypotension are shown in Table 3. Among the five patients who were administered vasopressors, phenylephrine was selected among four patients. The remaining one patient who had dilated cardiomyopathy required phenylephrine, ephedrine, and dopamine. This hypotensive episode was observed after induction of general anesthesia, after administration of nitroglycerin for rapid tocolysis, and after delivery. Serum creatinine remained unchanged after surgery compared with the preoperative level. Epidural catheters were withdrawn within 48 h of initial insertion. Postoperative complications such as infections, pneumonia, hemorrhage, embolic events, or acute kidney injury were not detected. Within 1 year post-surgery, there were no confirmed complications, such as kidney rejection, kidney degeneration, and complications from general or regional anesthesia.

**Table 2:** Surgical information

	Total (*n* = 14)	GA (*n* = 5)	RA (*n* = 9)
Operation time (min)	69 (22)	69 (26)	69 (19)
Intraoperative fluid volume (mL)	1267 (508)	1300 (799)	1249 (215)
Blood loss (g)	895 (463)	891 (483)	897 (451)
Intraoperative urine volume (mL)	371 (248)	230 (160)	450 (253)
Preoperative			
SAP (mmHg)	156 (21)	164 (26)	151 (17)
DAP (mmHg)	100 (16)	106 (13)	97 (17)
Intraoperative			
Maximum SAP (mmHg)	170 (20)	184 (17)	162 (17)
Maximum DAP (mmHg)	102 (15.4)	111 (2)	97 (17)
Minimum SAP (mmHg)	97 (13)	87.8 (11)	102 (11)
Minimum DAP (mmHg)	49 (12)	47 (13)	50 (10)
MAP < 65 mmHg or use of vasopressors	6 (43%)	3 (60%)	3 (33%)
Postoperative creatinine (mg/dL)	1.47 (0.52)	1.83 (0.54)	1.27 (0.37)
Postoperative AKI	0	0	0

Values are mean (SD) or number (%)

*GA* general anesthesia, *RA* regional anesthesia, *SAP* systolic arterial pressure, *DAP* diastolic arterial pressure, *MAP* mean arterial pressure, *AKI* acute kidney injury

**Table 3:** Data for patients with intraoperative MAP < 65 mmHg

Case	Anesthesia method	Timing of hypotension	Duration under MAP < 65 mmHg	Vasopressors (intraoperative total dose)
1	GA	Induction of GA, post-delivery	17.5 min	Phenylephrine (0.15 mg)
2	GA	Induction of GA, post-delivery	15 min	No use
3	GA	Induction of GA, after NTG infusion	13 min	Phenylephrine (0.3 mg), ephedrine (4 mg), and dopamine (1–3 μg/kg/min)
4	RA	After NTG infusion	3 min	Phenylephrine (0.45 mg)
5	RA	After NTG infusion	5 min	Phenylephrine (0.05 mg)
6	RA	After NTG infusion	2 min	Phenylephrine (0.3 mg)

*MAP* mean arterial pressure, *GA* general anesthesia, *NTG* nitroglycerin, *RA* regional anesthesia

The neonatal outcomes are shown in Table 4. A total of four patients (27%) had 1 min Apgar score of less than 7 (three general anesthesia and one regional anesthesia). Umbilical artery pH (UApH) was slightly higher among regional anesthesia patients (7.29 vs 7.24), but no case with UApH < 7.1 was observed in both groups.

**Table 4:** Neonatal outcomes

Neonatal number	13 singletons, 1 twin		
	Total	GA	RA
*N*	15	5	10
Gestational weeks	33.3 (4.1)	30.7 (3.6)	34.7 (3.6)
Weight at birth (g)	1853 (933)	1461 (915)	2050 (879)
Apgar 1 min < 7	4 (27%)	3 (60%)	1 (10%)
Apgar 5 min < 7	2 (13%)	2 (40%)	0 (0%)
UApH	7.27 (0.05)	7.24 (0.07)	7.29 (0.03)
NICU admission	11 (73%)	5 (100%)	6 (60%)

Values are mean (SD) or number (%)

*GA* general anesthesia, *RA* regional anesthesia, *UApH* umbilical artery pH, *NICU* neonatal intensive care unit

## Discussion

Among the 14 cases in this study, the rate of cesarean delivery and preterm delivery was 63% and 64%, which were twice and more than sixfold higher, respectively, compared with the general population in the USA [8, 9]. These results are consistent with previous studies [2, 3]. The cases in our study were older with younger gestational age compared with a previous study [6]. Although the period between renal transplant and cesarean delivery was shorter compared with previous reports [6], it was longer than 2 years, recommended as a safe period by both the USA and European Union guidelines [10, 11].

Most cases received a combination of immunosuppressants and corticosteroids during pregnancy. Although the incidence of epidural abscess caused by regional anesthesia in parturients after renal transplant remains unknown, patients receiving immunosuppressants are vulnerable to infections [12], suggesting that epidural abscess would be more commonly caused by neuraxial anesthesia in those women and strict aseptic techniques are recommended. We removed epidural catheters within 48 h in all patients in order to prevent infections following the suggestion by Gronwald [12].

According to a multicenter cohort study [6], indications for cesarean delivery after renal transplant are previous cesarean delivery (23%), material medical causes (23%), fetal causes (18%), obstetric delivery causes (15%), material preference (21%), and emergency surgery (20%). In the present study, HDP was the most common indication of cesarean delivery. HDP was observed in 57% in the present study, higher than 24% in the previous review [2]. This would result that the mean age of the study subjects was approximately 5 years older than in that review. The proportion of patients who received general anesthesia was 36%, higher than that reported in previous studies [6]. The choice of anesthesia method was at the discretion of the attending anesthesiologists, and there were various reasons for it. Because the incidence of cesarean delivery was high, sharing of information of post-renal transplant parturients would be important to prepare for anesthesia. In this study, post-renal transplant parturients scheduled for cesarean delivery visited the anesthesiology outpatient clinic twice: from 22 to 25 weeks and after 32 weeks. If the physical findings, renal function, and fetal growth information from the outpatient clinic are evaluated, the optimal anesthesia method can be planned and put into practice smoothly even in an emergency.

Specific points to be aware of for effective anesthesia management in cesarean delivery include circulatory management and maintenance of renal function. Prolonged operation time due to previous abdominal surgery should also be considered, although none of the cases required a transition of anesthesia method from regional to general during surgery. The highest systolic and diastolic blood pressure during surgery tended to be higher under general anesthesia than under regional anesthesia, which would be ascribed to a high proportion of patients with HDP. Mean arterial blood pressure < 65 mmHg which lasted for ≥ 13 min during general anesthesia increased the postoperative acute kidney injury in noncardiac surgery [13]. In the present study, this occurred in three patients under general anesthesia and in no patient under regional anesthesia. Our study confirmed that hypotension typically occurred after induction of general anesthesia, after administration of nitroglycerin, and after delivery. Although the kidney is in a different position and we cannot solely focus on achieving a mean arterial pressure ≥ 65 mmHg, prolonged hypotension should be avoided. Hypotension occurred after the administration of nitroglycerin in all patients irrespective of the anesthesia method. Because nitroglycerin is used for rapid tocolysis in preterm delivery, it would be more frequently used in post-renal transplant cesarean delivery. Although there were no cases of postoperative acute kidney injury in our study, prediction and treatment of hypotension to improve circulatory management are crucially important. Despite numerous reports comparing the effect of ephedrine and phenylephrine used for treating hypotension during cesarean delivery [14–18], there is no unified view regarding the agents for the treatment of hypotension, particularly in post-renal transplant patients. Urinary output must be monitored during surgery since transplanted kidneys can be physically compressed by the gravid uterus during pregnancy or during surgery, resulting in hydronephrosis or acute kidney injury [19, 20].

This was a single-institution retrospective study and is limited by an extremely small sample size. Since the details of how the anesthetic method was selected were not always available, in some cases, the reason behind the choice between general anesthesia and regional anesthesia was unknown. Furthermore, the small number of cases precludes any meaningful statistical analysis.

## Conclusions

This study provided specific details on the hemodynamics of cesarean delivery in post-renal transplant patients. In our hospital, many of the cesarean delivery for post-renal transplant patients were for preterm delivery and emergency surgery, with hypertensive disorders of pregnancy as the most common indication. During surgery, both hypertension and hypotension are likely to develop, leading to difficulties in managing circulation and requiring careful treatment, with particular focus on maintaining renal perfusion and postoperative renal function. While the parturients and fetuses in our study did not suffer any complications due to surgery or anesthesia management, such as postoperative acute kidney injury, kidney deterioration, or kidney rejection, the small number of cases in this study necessitates further research at multiple centers to produce more widely generalizable results.

## Data Availability

The datasets used and/or analyzed during the current study are available from the corresponding author on reasonable request.

## Abbreviations

HDP: Hypertensive disorders of pregnancy
UApH: Umbilical artery pH
